# Factors Associated with Unplanned Primary Cesarean Birth: Secondary Analysis of the *Listening to Mothers in California* Survey

**DOI:** 10.1186/s12884-020-03095-4

**Published:** 2020-08-14

**Authors:** Carol Sakala, Candice Belanoff, Eugene R. Declercq

**Affiliations:** 1 National Partnership for Women & Families, 1875 Connecticut Avenue, NW, Suite 650, Washington, DC 20009 USA; 2 Boston University School of Public Health, 801 Massachusetts Avenue Crosstown Center, 4th Floor, Boston, MA 02118 USA

**Keywords:** Cesarean birth, Cesarean rate reduction, Maternal agency, Labor management, Surveys of childbearing women, *Listening to Mothers* survey

## Abstract

**Background:**

In many countries, cesarean section has become the most common major surgical procedure. Most nations have high cesarean birth rates, suggesting overuse. Due to the excess harm and expense associated with unneeded cesareans, many health systems are seeking approaches to safe reduction of cesarean rates. Surveys of childbearing women are a distinctive and underutilized source of data for examining factors that may contribute to cesarean reduction.

**Methods:**

To identify factors associated with unplanned primary cesarean birth, we carried out a secondary analysis of the *Listening to Mothers in California* Survey, limited to the subgroup who had not had a previous cesarean birth and did not have a planned primary cesarean (n = 1,964). Participants were identified through birth certificate sampling and contacted initially by mail and then by telephone, text message and email, as available. Sampled women could participate in English or Spanish, on any device or with a telephone interviewer. Following bivariate demographic, knowledge and attitude, and labor management analyses, we carried out multivariable analyses to adjust with covariates and identify factors associated with unplanned primary cesarean birth.

**Results:**

Whereas knowledge, attitudes, preferences and behaviors of the survey participants were not associated with having an unplanned primary cesarean birth, their experience of pressure from a health professional to have a cesarean and a series of labor management practices were strongly associated with how they gave birth. These practices included attempted induction of labor, early hospital admission, and labor augmentation. Women’s reports of pressure from a health professional to have a primary cesarean were strongly related to the likelihood of cesarean birth.

**Conclusions:**

While women largely wish to avoid unneeded childbirth interventions, their knowledge, preferences and care arrangement practices did not appear to impact their likelihood of an unplanned primary cesarean birth. By contrast, a series of labor management practices and perceived health professional pressure to have a cesarean were associated with unplanned primary cesarean birth. Improving ways to engage childbearing women and implementing changes in labor management and communication practices may be needed to reduce unwarranted cesarean birth.

## Background

In many countries, cesarean section is the most common major surgical procedure. Globally, the rate of cesarean birth increased by 3.7% annually from 2000 to 2015. Exceptionally wide practice variation characterizes use of cesarean birth both across and within countries. Cesarean rates of less than 10%, considered an indication of underuse, have recently been identified in 28 nations. However, most world nations have cesarean rates of 15% or higher, which is considered an indication of overuse [1]. As overuse is associated with myriad types of excess harm in women and cesarean-born children and with excess cost, policymakers, purchasers, payers, clinical leaders, researchers, advocates and women themselves seek ways to minimize unneeded cesarean births [2, 3].

In the United States, following a steep rise between 1996 and 2007, the cesarean rate has plateaued for a decade at nearly one in three. The American College of Obstetricians and Gynecologists and Society for Maternal-Fetal Medicine concluded that the steep rise was not accompanied by discernible benefits for women and newborns, and they have jointly issued guidance for safely reducing primary cesarean births [4].

Many effective strategies for cesarean reduction have been identified. Pre-labor practices that have been associated with reduced likelihood of cesarean birth include choosing individual care providers and birth settings with lower cesarean rates, choosing types of care providers and birth settings with lower cesarean rates (e.g., midwives and birth centers), childbirth training workshops, being physically active and staying fit during pregnancy, arranging for the labor support of a doula and attempting external cephalic version with non-cephalic presentation at term [4–8]. After the onset of labor, factors that have been associated with reduced likelihood of cesarean birth include: delaying hospital admission until labor is well established, continuous labor support by someone in a doula role, intermittent auscultation rather than either on-admission or continuous electronic fetal monitoring, discontinuation of synthetic oxytocin for induction after onset of labor, avoiding labor epidural (associated with cesarean for non-reassuring fetal heart status), remaining upright and mobile during the first stage of labor versus lying in bed, and following guidelines related to cervical status and elapsed time for use of synthetic oxytocin and cesarean birth [7, 9–14]. Clinician interventions associated with lower likelihood of cesarean birth include clinical practice guidelines coupled with education by opinion leaders, audit and feedback, or mandatory second opinion [3, 15, 16].

Characteristics of women associated with lower likelihood of cesarean birth include lower body mass index, younger age and having Medicaid/public assistance versus private insurance coverage [17–20]. Cesarean rates vary across racial/ethnic groups, and further within specific ethnic sub-groups [21–23]. Changes over time in primary cesarean rates have not been associated with changes in maternal risk profiles [24].

Assessment of factors that may be associated with cesarean rates commonly use vital statistics, administrative and/or medical records as data sources. Surveys of childbearing women themselves enable us to examine the possible impact of women’s beliefs and behaviors, as well as their care arrangements and labor experiences that may not be systematically recorded in other sources [25, 26]. Here we use data from the recent population-based *Listening to Mothers in California* Survey to examine mother-reported factors that may be associated with lower likelihood of cesarean birth. These include factors not previously reported in the literature, such as women’s attitudes, behaviors, knowledge and preferences, as well as previously reported factors, such as members of the woman’s care team and labor management practices. We also used the low-risk first birth cesarean rate at the woman’s birth hospital as a proxy for local practice style [27]. To contribute to discussions about optimal ways to safely reduce the rate of cesarean birth, we carried out an adjusted analysis to examine possible associations between women’s likelihood of cesarean birth and their attitudes, behaviors, knowledge and preferences, as well as their experiences with health professionals, the composition of their care team, and labor management and communication practices they experienced.

## Methods

The *Listening to Mothers in California* Survey was developed through a collaboration of investigators from the National Partnership for Women & Families, Boston University School of Public Health and University of California, San Francisco (UCSF) Center on Social Disparities in Health, who worked with Quantum Market Research to plan and carry out the survey. The sampling frame for the *Listening to Mothers* in *California* study was drawn from California birth certificate data for births between September 1 and December 15, 2016. We excluded women less than 18, women with out-of-hospital births, women with multiple births and non-residents of California. Further exclusions during the field period included women who could not participate in English or Spanish and women who were not currently living with their baby. We oversampled Black women, women with midwifery-attended births and those with vaginal births after cesarean to increase power for better understanding the experiences and perspectives of women within these smaller groups. The field period was from February 22 through August 15, 2017. Sampled women were invited to participate through an initial and – as needed – three reminder mailings with distinctive envelopes and two inserts designed to be engaging and stand apart from typical residential mail. The mailings included a cover letter inviting participation and incorporating elements of informed consent and an insert with information about how to participate in the survey. The survey research firm contacted sampled women who did not respond to mailings by – as available from additional sources of information – email, text message and telephone calls. Sampled women could choose among multiple ways to participate: online on their own using any device or by telephone with a trained interviewer. Participants were from 2 to 11 months postpartum when they responded. Among all participants, 81% selected the English version of the questionnaire, and 19% selected the Spanish version. Among all participants, 34% completed the survey online, 28% completed by phone with an interviewer and 39% completed using both methods (a common pattern was starting on their own online and finishing later with an interviewer).

Both outreach materials inviting sampled women’s participation and the survey questionnaire (see Additional File) were developed to address population and policy issues relevant to the state of California. The questionnaire includes some items that continue verbatim from national *Listening to Mothers* surveys, some items adapted from those previous surveys (included for mobile-first suitability, enabling participants to complete the survey on a smartphone or tablet), and some items newly developed for or included with permission in the California survey. Initial English versions of outreach materials and questionnaire were pilot tested with varied populations who had recently given birth and refined over several iterations. Subsequently, these materials were translated into Spanish (with back translation) and adjusted accordingly, followed by iterative testing and refinement with varied groups of Spanish-speaking childbearing women. The survey took a bit longer than a half-hour to complete on average. The *Listening to Mothers in California* full survey report and related materials, including survey questionnaire, are available at websites of both the sponsor [28] and funder [29].

UCSF analysts used demographic and other relevant variables from the statewide 2016 Birth Statistical Master File to weight the final sample to be closely representative of childbearing women aged 18 and older giving birth to single babies in California hospitals throughout 2016. Our response rate calculation was based on methods of the American Association of Public Opinion Research, using their “Response Rate 2” and “Response Rate 4” methods, which exclude sampled participants found to be ineligible during the field period (e.g., baby not living with mother or respondent unable to participate in English or Spanish) and which further estimates and excludes the proportion of sampled women of unknown eligibility who were ineligible. This resulted in a response rate of 55% for the final sample size of 2,539 women.

Appendix B in our *Listening to Mothers in California* full survey report compares a series of demographic variables in statewide birth certificate data for women 18 + with singleton births and in our weighted dataset. The discrepancies between the state and our dataset are overwhelmingly in the range of 0 to 2 percentage points. For example, age 18–19 (3% state, 4% dataset), age 35+ (22%, 22%), Latina (48%, 50%), Asian/Pacific Islander (16%, 16%), Black (5%, 5%), US-born (63%, 65%), first birth (38%, 40%), less than high school education (13%, 11%), certified nurse-midwife birth attendant (10%, 9%), cesarean birth (31%, 30%), preterm birth (7%, 7%), very low birth weight (1%, 1%), and low birth weight (4%, 5%) [28]. The report appendices present a more detailed explanation of the methodology [28].

The Committee for the Protection of Human Subjects, within California’s Office of Statewide Health Planning and Development, is the institutional review board (IRB) of record for the *Listening to Mothers in California* Survey. This Committee determined that the study posed a low risk to participants and approved the study and several protocol amendments. The UCSF IRB also approved the project. The California Department of Public Health Vital Statistics Advisory Committee approved and provided access to birth certificate data for sampling, contacting sampled women, data weighting and data analysis. Given the rich array of health insurance options available in California, Department of Health Care Services analysts linked birth certificate items to the Management Information System/Decision Support System Warehouse to definitively identify participants with Medi-Cal (California’s Medicaid public assistance program) coverage of their childbirth, defined as Warehouse evidence of a paid claim for a 2016 birth. In this analysis, privately insured women did not have such a claim and identified a source of private insurance in survey responses.

We used publicly available 2016 data to classify the nulliparous, term, singleton, vertex (NTSV) cesarean rate of respondents’ birth hospitals into four quartiles and included those data in the multivariable analysis as a proxy for local institutional culture associated with the likelihood of a cesarean birth. This first-birth low-risk (“NTSV”) cesarean rate is widely used in the United States as a measure that is case-mix adjusted and thus better suited to comparisons among facilities than total or primary cesarean rates.

In this report, Latina indicates participants who self-identified as “Hispanic or Latina.” White, Asian and Pacific Islander, and Black reference participants who did not choose “Hispanic or Latina” and self-identified, respectively, as white, Asian or Native Hawaiian or other Pacific Islander, and Black. There was some apparent uncertainty over the interpretation of the term and questionnaire descriptions of “doula” among Spanish speaking respondents and/or women who did not primarily speak English in their homes, and therefore the data presented on doula responses are limited to those who primarily spoke English in their homes.

We limited the present secondary analyses to women whose index birth was an unplanned primary cesarean (excluding women with planned primary cesareans that occurred before the onset of labor) and women who did not have a history of cesarean birth and gave birth vaginally. Thus, participants with a 2016 vaginal birth after cesarean or repeat cesarean were excluded. In our full survey sample, 11% of participants had an unplanned primary cesarean, 5% had a planned primary cesarean and 15% had a repeat cesarean [30].

We conducted bivariate analyses, examining associations between selected maternal socio-demographic characteristics (including age, race/ethnicity, insurance, parity, body mass index (BMI), marital status, nativity, language used at home and education level) and mode of birth. We also examined the association between various maternal knowledge, beliefs and preferences relating to the intrapartum experience, and selected labor management experiences, and the risk of an unplanned primary cesarean birth. All but the measures of pregnancy complications, labor and birth risks and gestational age were drawn from respondent’s answers on the survey. Gestational age (< 37 weeks or 37 + weeks), any pregnancy complications (e.g., asthma, diabetes, hypertension, exclusive of prenatal screening) and any labor and delivery risks (e.g., meconium staining, abruptio placenta, prolapsed cord, premature rupture of membranes) were drawn from the respondent’s birth certificate. While the validity of the measurement of some of the individual complications on the birth certificate may be problematic [31], identifying women with none of these items reported identifies a lower risk subset of the study sample.

The final, fully-adjusted model examined the risk of unplanned cesarean birth and included variables chosen a priori either because they were statistically significantly related in the bivariate analysis or they have been shown to be related to cesarean births in the past. They were divided into sociodemographic (race/ethnicity, maternal age, education, parity and type of insurance), health (BMI, pregnancy complications, labor and birth risks and gestational age), maternal attitudinal and experiential (feelings about medical interference with labor and sense of being pressured to have a cesarean) and pregnancy and labor management variables (prenatal provider, attempted induction, dilation at hospital admission, augmentation after labor began, amniotomy after labor began). Finally, as a surrogate for maternity unit culture, a hospital-level measure of the NTSV cesarean birth rate was included [27]. Because of the strong association between parity and reports of pressure to have a cesarean birth, we further stratified the full multivariable model by parity (nulliparous versus multiparous).

## Results

There were 1,964 respondents in our sample who had not had a previous cesarean birth and whose current birth was either vaginal or an unplanned cesarean. We examined possible variation in experience of unplanned primary cesarean birth within demographic categories (Table 1). Experiencing an unplanned primary cesarean varied by race/ethnicity and parity, with Latina (12.8%) and white women (13.8%) less likely than Black women (25.5%), and women with one (4.5%) or more (6.3%) prior vaginal births far less likely than first-time mothers (23.7%) to have a primary cesarean.

**Table 1:** Demographic Distribution and Unplanned Primary Cesarean Birth among Women without a Prior Cesarean, California, 2016

Category	Proportion^a^	Unplanned Primary Cesarean
	(*n* = 1,964) %	**% (95% C.I.)**
ALL		14.0 (12.5, 15.7)
Mother’s Age		
<25	23.9	11.9 (9.2, 15.2)
25–29	26.9	15.5 (12.5, 19.0)
30–34	29.2	15.6 (12.7, 19.0)
35+	20.0	14.2 (10.8, 18.5)
Mother’s Race/Ethnicity		
White	27.4	13.8 (10.9, 17.3)
Black	4.4	**25.5 (19.2, 33.0)**
Asian and Pacific Islander	16.0	12.8 (10.7, 15.1)
Latina	48.8	16.7 (12.5, 22.0)
Insurance		
Medi-Cal	47.0	14.2 (12.0, 16.7)
Private	48.3	14.1 (12.0, 16.7)
Parity		
1	47.4	**23.7 (21.0, 26.6)**
2	28.8	4.5 (3.0, 6.8)
3+	23.8	6.3 (4.3, 9.1)
Body Mass Index		
Underweight	16.1	13.2 (9.6, 17.9)
Normal	44.0	13.8 (11.4, 16.6)
Overweight	22.6	11.1 (8.3, 14.8)
Obese	17.2	17.6 (13.6, 22.5)
Marital Status		
Married	59.3	13.9 (11.9, 16.2)
Living with someone	27.0	12.4 (9.8, 15.7)
Single	12.0	19.3 (14.7, 24.9)
Birthplace		
US	66.2	15.2 (13.2, 17.3)
Other country	33.8	12.4 (9.9, 15.4)
Language at Home		
English only	58.4	15.2 (13.1, 17.4)
Spanish only	15.5	9.6 (6.8, 13.5)
Asian language	7.8	17.4 (11.4, 25.5)
Education		
High school or less	31.3	11.6 (9.2, 14.5)
Some college	33.0	15.8 (13.1, 19.0)
College	19.7	13.7 (10.4, 17.8)
Some grad school or higher	16.1	16.7 (12.9, 21.4)

**Bold** indicates significant differences from the overall average at *p* < .05

^a^Figures do not always total 100% due to rounding and exclusion of minor categories

Table 2 presents the unadjusted prevalence of cesarean birth associated with women’s knowledge, beliefs and preferences. Primary cesarean rates trended toward being higher, among women who agreed versus disagreed in the postpartum period that “childbirth is a process that should not be interfered with unless medically necessary,” though the differences did not reach significance. Having sought cesarean rates of prospective hospitals for giving birth was not associated with the likelihood of having a primary cesarean, nor was knowledge about variation in quality among obstetricians and among hospital maternity units (versus believing quality does not vary or not being sure). Interest in having a midwife, or having a doula, should the woman give birth in the future was also not associated with having had a primary cesarean. Only interest in having a future birth center birth differed: women who had had a primary cesarean were less likely to be interested in a future birth center birth and more likely to either not want or be unsure about a future birth center birth.

**Table 2:** Unplanned Primary Cesarean Birth by Maternal Beliefs among Women Planning a Vaginal Birth, California, 2016

Category	Proportion	Unplanned Primary Cesarean
	(*n* = 1,964) %	**% (95% C.I.)**
Maternal Attitude: *Childbirth is a process that should not be interfered with unless medically necessary*		
Agree	84.3	14.2 (12.4, 16.1)
Disagree	7.8	7.4 (4.1, 12.9)
*Did you try to learn about hospital cesarean rates?*		
Yes, and found it	25.9	14.6 (11.7, 18.1)
Yes, and did not find it	4.5	13.1 (7.5, 22.1)
No, did not look	69.7	14.0 (12.2, 16.0)
*Is the quality of all obstetricians the same in your area?*		
Yes, pretty much the same	32.2	15.7 (12.9, 19.0)
Not Sure	34.7	13.9 (11.3, 16.9)
No, big differences	33.1	13.0 (10.6, 15.8)
*Is the quality of all hospital maternity services the same in your area?*		
Yes, pretty much the same	35.8	15.8 (13.2, 18.9)
Not Sure	34.9	13.6 (11.0, 16.8)
No, big differences	29.4	13.0 (10.6, 15.8)
*Interest in a midwife in the future*		
Definitely want or would consider	56.4	13.4 (11.5, 15.7)
Definitely do not want or not sure	43.5	15.1 (12.8, 17.8)
*Interest in a doula in the future*		
Definitely want or would consider	58.8	13.7 (11.8, 15.9)
Definitely do not want or not sure	41.2	14.8 (12.3, 17.6)
*Interest in a birth center in the future*		
Definitely want or would consider	41.2	11.3 (9.2, 13.8)
Definitely do not want or not sure	58.8	**16.2 (14.1, 18.6)**

**Bold** indicates significant differences within the category at *p* < .05

Table 3 examines possible variation in mode of birth associated with women’s experiences with maternity care personnel and with labor management practices. Those who reported experiencing pressure from a health professional to have a cesarean were far more likely to have a primary cesarean (54% versus 11%). However, experiencing pressure for induced labor or an epidural was not associated with having a primary cesarean birth. Also not associated with having a primary cesarean birth were having had a midwife as one’s predominant prenatal care provider, having had a labor doula, and three staff support measures: whether the woman agreed that while she was giving birth, staff had encouraged her to make decisions, she felt well supported by staff and staff had communicated well with her. A number of labor management practices were associated with having an unplanned primary cesarean. These included having a cervical dilation of less than 5 centimeters versus 5 or more centimeters at the first vaginal exam after hospital admission (17% versus 7%), having experienced attempted labor induction (19% versus 9%), experiencing use of synthetic oxytocin to stimulate established labor (17% versus 6%), experiencing epidural analgesia (18% versus 3%) and being upright or mobile (versus in bed) during labor (14% versus 10%). Labor management practices not associated with experiencing an unplanned primary cesarean were: labor induced with amniotomy, amniotomy after the start of labor and use of any intermittent auscultation. Because we did not know about the timing of epidural analgesia and could not identify women who experienced this procedure as a pain relief method for surgery or in anticipation of surgery, we do not further include this variable in our analysis.

**Table 3:** Unplanned Primary Cesarean Birth and Selected Labor Management Experiences, California, 2016

Variable	Proportion (*n* = 1,964) %	Unplanned Primary Cesarean among women without condition	Unplanned Primary Cesarean among women with condition
Gestational age > = 37 weeks (Full Term), cephalic presentation	94.7	21.1 (13.9, 30.7)	12.4 (10.9, 14.1)
**Care Arrangements and Support**			
Had a midwife for prenatal care	8.4	14.3 (12.7, 16.1)	11.8 (7.7, 17.9)
Had a doula in labor (English speaking only)	9.5	13.3 (8.1, 20.9)	15.0 (12.8, 17.5)
Felt pressure from any health professional to have an induction	16.4	16.9 (13.0, 21.8)	13.5 (11.9, 15.3)
Felt pressure from any health professional to have an epidural	11.7	17.7 (13.1, 23.5)	13.6 (12.0, 15.3)
Felt pressure from any health professional to have a cesarean	8.1	**10.5 (9.1, 12.0)**	**53.9 (45.6, 61.9)**
Agreed staff encouraged her to make decisions	75.6	13.9 (11.0, 17.5)	10.6 (9.1, 12.4)
Agreed well supported by staff when giving birth	92.0	15.2 (10.2, 21.9)	11.2 (9.7, 12.8)
Agreed staff communicated well with during labor	91.9	13.3 (8.8, 19.5)	11.3 (9.9, 12.9)
**Labor Management**			
5 + cm dilation first vaginal exam after admission	23.8	**17.1 (14.9, 19.5)**	**6.6 (4.2, 10.1)**
Medical induction attempted	47.7	**9.4 (7.7, 11.4)**	**19.2 (16.7, 21.9)**
Induced with amniotomy	12.4	20.1 (17.1, 23.4)	16.7 (12.4, 22.2)
Amniotomy after labor began	45.5	12.8 (10.8, 15.2)	10.7 (8.8, 13.1)
Used any intermittent auscultation	19.4	11.2 (9.6, 13.1)	9.6 (4.4, 19.7)
Synthetic oxytocin to stimulate after labor began	42.3	**5.7 (4.3, 7.4)**	**16.8 (14.3, 19.7)**
Epidural	71.7	**3.4 (2.2, 5.3)**	**18.2 (16.2, 20.4)**
Mobile during labor	38.0	**9.7 (8.1, 11.6)**	**14.1 (11.7, 17.0)**
NTSV rate above the target (23.9%)	54.3	12.1 (10.1, 14.5)	15.9 (13.7, 18.3)

In the fully-adjusted analysis, we included variables based on theoretical or statistical significance. Covariates in this model included all those listed in Table 4: maternal race/ethnicity, age, education, parity, pre-pregnancy BMI, insurer, maternal attitude toward birth, prenatal provider, attempted induction, gestational age, dilatation at admission, mobility during labor, stimulation of labor, amniotomy, any pregnancy or labor and birth complication (reported on birth certificate), and overall low-risk hospital cesarean rate. In this multivariable model (Table 4), women who were Black had over twice the risk of unplanned primary cesarean compared to white women, as did women with less than a college degree compared to those with a college degree or higher. Women who were between 18 and 24 years of age at birth had less than half the risk of an unplanned primary cesarean compared to older women, while women who had given birth vaginally in the past had an 88% lower risk. Women who reported feeling pressure from a health professional to have a cesarean were more than 7 times more likely to have had one. While an attitude favoring less intervention in childbirth was suggestive of a lower risk of unplanned cesarean, it did not achieve significance.

**Table 4:** Risk of Unplanned Primary Cesarean Birth, Fully Adjusted Model, Overall and by Parity, California, 2016^a^

Variable	Adjusted Odds Ratio^b^ (95% confidence interval) (*n* = 1,964)	Adjusted Odds Ratio (95% confidence interval) Stratified: Parity 1 (*n* = 974)	Adjusted Odds Ratio (95% confidence interval) Stratified: Parity 2+ (*n* = 990)
**DEMOGRAPHICS**			
* Race/Ethnicity*			
White (ref)			
Asian	1.29 (0.68, 2.44)	0.93 (0.46, 1.90)	6.87 (0.97, 48.49)
Latina	1.13 (0.68, 1.87)	1.09 (0.64, 1.86)	3.61 (0.72, 18.01)
Black	**2.40 (1.31, 4.42)**	**2.61 (1.35, 5.02)**	3.82 (0.39, 37.56)
* Mother’s Age*			
18–24	**0.49 (0.27, 0.88)**	**0.41** (**0.22, 0.78)**	0.70 (0.12, 3.93)
25–29 (ref)			
30–34	1.20 (0.69, 2.06)	1.06 (0.57, 1.98)	1.48 (0.44, 5.02)
35+	1.09 (0.64, 1.86)	0.83 (0.43, 1.59)	1.92 (0.40, 9.18)
* Mother’s Education*			
HS or less	**2.30 (1.10, 4.80)**	**2.52** (**1.14, 5.58)**	0.83 (0.14, 4.94)
Some College	**2.31 (1.31, 4.07)**	**2.24** (**1.25, 4.03)**	1.25 (0.30, 5.14)
College grad (ref)			
Graduate degree	1.56 (0.82. 3.00)	1.63 (0.86, 3.07)	0.69 (0.10, 4.68)
* Parity*			
1 (ref)			
2+	**0.12 (0.07, 0.20)**	**---**	**---**
* Insurance Payer*			
Medi-Cal (ref)			
Private	1.22 (0.97, 1.97)	1.04 (0.58, 1.89)	3.11 (0.79, 12.34)
* Body Mass Index*			
Underweight	0.89 (0.50, 1.58)	0.76 (0.40, 1.45)	3.88 (0.70, 21.67)
Normal (ref)			
Overweight	1.01 (0.63, 1.62)	0.82 (0.47, 1.41)	2.29 (0.50, 10.44)
Obese	1.23 (0.73, 2.08)	1.27 (0.68, 2.37)	1.27 (0.37, 4.35)
**MATERNAL ATTITUDE**			
* Childbirth shouldn’t be interfered with*			
Don’t strongly agree (ref)			
Strongly agree	0.73 (0.50, 1.08)	0.69 (0.45, 1.07)	1.02 (0.30, 3.42)
* Felt Pressure to have a Cesarean*			
No (ref)			
Yes	**7.45 (4.23, 13.1)**	**5.29** (**2.72, 10.28)**	**41.84** (**11.30, 154.88**)
**CARE TEAM, MANAGEMENT**			
* Prenatal Provider*			
Obstetrician (ref)			
Midwife	1.00 (0.54, 1.88)	1.01 (0.50, 2.04)	0.99 (0.09, 10.58)
* Attempted Induction*			
No (ref)			
Yes	**2.07 (1.33, 3.21)**	**1.75** (**1.07, 2.85**)	**5.05** (**1.29, 19.76**)
* Gestational Age*			
< 37 weeks (ref)			
37 + weeks	0.77 (0.35, 1.71)	0.75 (0.34, 1.67)	0.84 (0.01, 55.05)
* Dilation at Admission*			
< 5 centimeters	**2.85 (1.09, 7.47)**	**5.53** (**1.37, 22.29**)	0.65 (0.16, 2.69)
5 + centimeters (ref)			
* Mobile during Labor*			
No	0.73 (0.51, 1.05)	0.85 (0.57, 1.26)	0.28 (0.08, 1.03)
Yes (ref)			
* Synthetic Oxytocin to Stimulate after Labor Began*			
No (ref)			
Yes	**1.74 (1.08, 2.79)**	1.61 (0.96, 2.72)	1.92 (0.59, 6.28)
* Amniotomy after Labor Began*			
No (ref)			
Yes	**0.61 (0.44, 0.87)**	**0.63** (**0.42, 0.95**)	0.52 (0.17, 1.59)
* Pregnancy Complications*			
No (ref)			
Yes	0.84 (0.49, 1.43)	1.04 (0.58, 1.86)	0.47 (0.13, 1.65)
* Labor & Delivery Complications*			
No (ref)			
Yes	1.77 (0.98, 3.23)	1.50 (0.77, 2.95)	2.82 (0.54, 14.65)
* Hospital NTSV*^*c*^ *Rate*			
Quartile 1 (ref)			
Quartile 2	1.02 (0.56, 1.87)	1.09 (0.55, 2.17)	0.69 (0.16, 2.92)
Quartile 3	1.09 (0.65, 1.82)	1.03 (0.57, 1.84)	1.00 (0.34, 2.98)
Quartile 4	1.53 (0.90, 2.58)	1.26 (0.65, 2.42)	1.95 (0.56, 6.86)

^a^ In the cases of race, body mass index, prenatal provider, and dilation at admission, small numbers of “other” cases were not presented

^b^ This model controls for parity and other covariates identified in the text

^c^ NTSV – cesarean rate among nulliparous women giving birth at term with singleton in vertex presentation

**Bold** – significant at *p* < .05 level

Overall, women who experienced an attempted induction were twice as likely to report an unplanned cesarean as those without, and those who were less than 5 centimeters dilated at admission were almost 3 times as likely to have a cesarean compared to those 5 or more centimeters dilated. Women reporting that their labor was augmented with Pitocin were also twice as likely to experience a cesarean while it appears amniotomy after labor began was protective. In the model including hospital-level NTSV cesarean rate presented in quartiles, NTSV-cesarean rate was not significantly related to a women’s likelihood of an unplanned primary cesarean.

In the stratified multivariable models, most significant relationships were confined to the nulliparous mothers. Significant differences in terms of race, mother’s age, mother’s education, amniotomy and admission at dilatation less than 5 centimeters were all limited to nulliparous mothers. Experiencing an attempted induction and feeling pressure to have a cesarean birth were significantly associated with elevated risk of cesarean in both groups, but more so in the multiparous group. However, these numbers should be interpreted with caution as the absolute number of multiparous mothers with a primary cesarean was small (*n* = 52).

## Discussion

Using data from the population-based *Listening to Mothers in California* Survey of women who had hospital births in 2016, we further analyzed the 1,964 women without a prior cesarean birth who did not have a planned cesarean birth. Our goal was to identify factors associated with reduced likelihood of unplanned primary cesarean birth using the broader range of variables available in woman-reported survey data. We examined in bivariate analyses the role of demographic variables; of women’s behaviors, preferences, knowledge and beliefs; and of characteristics of intrapartum care, including labor management practices. We then entered significantly related or theoretically important variables into multivariable analyses. While women’s personal views, preferences and behaviors related to care arrangement had little relationship with their likelihood of cesarean birth, we identified many aspects of their intrapartum care that were related to their mode of birth.

The use of survey data to understand factors associated with cesarean birth enabled us to explore whether the beliefs, preferences, knowledge and behaviors of women themselves play a role in their likelihood of having a cesarean. We found that most survey participants agreed either strongly (47%) or somewhat (27%) with the statement that “childbirth is a process that should not be interfered with unless medically necessary,” while just 8% disagreed. And although few actually used midwifery care and had doula support, and all gave birth in hospitals, they expressed a high degree of interest (i.e., they would definitely want or would consider) in these high-touch, low-tech forms of care should they give birth in the future: midwife (56%), doula (59%) and birth center births (41%). Further, almost one respondent in three (30%) sought information about cesarean rates of prospective hospitals, with the great majority of these finding this information. And about one in three correctly understood that the quality of care varies across both obstetricians and hospital maternity units [28]. However, we found no association between having a cesarean and agreeing that unneeded childbirth interventions should be avoided, seeking cesarean rates of prospective facilities, understanding that quality varies by obstetrician and by facility, or feeling that the intrapartum staff had accorded autonomy. Women with a cesarean were less likely to be interested in a future birth center birth, possibly because they correctly understood that many care providers encourage hospital birth and repeat cesareans for women with a previous cesarean. It is not possible to take women’s own perspectives into account in analyses limited to administrative, clinical or vital records data sources.

Engaging patients and their family members and caregivers in their health care is a growing policy priority, as reflected in the United States National Quality Strategy’s priority of “Ensuring that each person and family is engaged as partners in their care” [32]. However, our results suggest that women’s behaviors relating to making care arrangements and their preferences and views have virtually no impact on whether they experience an unplanned primary cesarean, and are no match for the institutional care practices they encounter. A consensus *Blueprint for Advancing High-Value Maternity Care* recommends many resources and supports to foster engagement of childbearing women during pregnancy, childbirth and the postpartum period. These include better performance measures and user-friendly, evidence-based public reporting of results of performance measurement. Currently, there are no woman-reported nationally-endorsed measures of the experience or outcomes of maternity care. There are no endorsed measures of care coordination or shared decision making in maternity care, nor any endorsed measures of physiologic childbirth or vaginal birth after cesarean.

Development and routine use of high-quality, evidence-based, up-to-date decision aids would also provide essential support to childbearing women. Care navigators can help women understand how to find and interpret comparative performance information, develop care plans, work through decision aids, and complete surveys to collect information about their experience and outcomes of care [33]. Also foundational are women’s access to basic informational resources about cesarean birth [34, 35], including the My Birth Matters campaign in the state where this survey was conducted [36]. Creating and reliably using such resources and supports might help close the gap between the care many women desire and the care they receive. It may be unrealistic to expect childbearing women to be drivers of higher-value maternity care and of achieving many of their own care goals without implementing more substantial ways of supporting their engagement in their care.

By contrast, we identified demographic characteristics, provider behaviors and labor management practices that were clearly associated with mode of birth. The finding of higher cesarean rates for Black mothers is consistent with prior research [37–39], though the differences identified here (aOR 2.40) after adjustment are greater than in those studies. Likewise, the finding of higher cesarean rates for older [40] and less educated mothers has been regularly confirmed [41]. The positive relationship between age and cesarean birth appears to be well-established [18]. However, whether some older women experience avoidable cesareans due to age-related professional expectations, and whether the other subgroups are experiencing biased care and a greater burden of iatrogenic harm are a priority for future research, given the growing recognition that structural racism and both explicit and implicit bias adversely impact the life circumstances, health and quality of care of Black and other disadvantaged groups [42–44]. Providing higher quality care to women from these groups may offer important opportunities for safely reducing the cesarean rate. As hospital maternity units examine the impact of their highly variable unit culture [27] on cesarean rates and other birth outcomes, ensuring respectful, evidence-based care for all women can improve birth outcomes, reduce disparities and reduce costs.

Type of insurance payer, while trending toward significance with higher rates for women with private insurance, did not achieve statistical significance. An earlier study likewise found differences by payer type in unadjusted rates, but no differences after adjustment [45]. More importantly, we identified a number of modifiable labor management practices that were related to the likelihood of an unplanned primary cesarean, specifically attempted induction, labor augmentation and early admission to the hospital [12]. We included as a covariate in our adjusted analysis any pregnancy or labor and birth complication on the participant’s birth certificate to adjust for a factor that might have influenced both these labor practices and cesarean mode of birth.

Our results suggest that opportunities currently exist for reducing cesarean use through broad implementation of evidence-based labor management practices. The *Blueprint for Advancing High-Value Maternity Care* identifies payment and delivery system reform, performance measurement and accountability, consumer engagement, movement toward interprofessional education and team-based care, attention to workforce composition and distribution, and filling priority research gaps as promising strategies for maternity care transformation and reliable delivery of appropriate care for childbearing women and newborns [33].

Consistent with other research [12], we found increased likelihood of cesarean birth with early versus delayed hospital admission (aOR 2.85) among the 68% of our study sample who could recall cervical dilation at their first vaginal exam after hospital admission. The relationship was limited to nulliparous mothers (aOR 5.53). As just 24% of women had initial dilation of 5 or more centimeters, delayed admission appears to be an underutilized practice for cesarean reduction.

Some studies have found midwifery care to be associated with reduced likelihood of cesarean birth [14]. As we only collected the type of intrapartum provider who delivered the baby, we could not examine the role of the type of maternity care provider during labor before any decision for an unplanned, physician-attended primary cesarean. Having a midwife as the primary prenatal care provider was not associated with reduced likelihood of unplanned primary cesarean. The exclusion of women with a planned primary cesarean and women with a history of cesarean in this secondary analysis appears to have masked differences between midwifery and obstetrical care. With our entire survey sample, we found that women with obstetrician-led prenatal care were more likely to have a cesarean birth (32%) than women with midwifery-led prenatal care (18%) (*p* < .01). When we further limited the comparison to lower-risk first-birth (NTSV) cesareans, notable differences persisted: 28% with obstetrician-led care versus 17% with midwifery-led care (*p* < .01). Prenatal provider differences were also great when we looked at vaginal birth after cesarean rates, which were 14% among women with obstetrician-led care versus 33% among women with midwifery-led care (*p* < .02) [30].

The relationship between attempted labor induction and cesarean birth is uncertain. Although the large ARRIVE trial found reduced cesarean birth with routine induction at 39 weeks [46], questions have been raised about this trial’s external validity and relevance to other populations and care settings and practices [47, 48]. Our survey-based results found that women with attempted labor induction were more likely to have an unplanned primary cesarean than women with spontaneous onset of labor (aOR 2.07). Similarly, the relationship between epidural analgesia and cesarean birth has been controversial. Unfortunately, our data do not enable us to distinguish between epidural use in labor that may have contributed to an unplanned cesarean compared to epidural use as an anesthetic for a cesarean procedure, further complicated by the practice of encouraging epidural placement if a cesarean might be anticipated. Hence, we did not include this practice in the multivariable model that is presented. The relationship between labor augmentation with synthetic oxytocin and cesarean birth has also been variable [49]. We found that such labor augmentation was associated with having an unplanned primary cesarean.

Some of our labor management results differ from best available evidence. Intermittent auscultation versus continuous electronic fetal monitoring has been found to be associated with vaginal birth [9]. However, just 3% of survey participants said they had been monitored solely with a handheld device. The present analysis was based upon those with any intermittent auscultation, most of whom also used electronic fetal monitoring (16% in our study population). While the direction of effect was as expected, the difference was small and a much larger sample would have been necessary to explore this difference, and especially use of intermittent auscultation alone, in a multivariable model. We were puzzled to find in the bivariate relationship that women who reported being upright and ambulatory for some period of time during labor versus laboring in bed after hospital admission were *more* likely to have an unplanned cesarean, but that relationship did not continue in the multivariable analysis [11]. Best evidence suggests that labor support in a doula role reduces the likelihood of cesarean birth [7], though our analysis found no difference in cesarean rates between women who did and did have labor support. Some of the discrepancy may be explained by the fact that we excluded women with previous cesareans, who may seek labor doulas to help achieve their goal of a vaginal birth after cesarean.

The largest adjusted odds ratio in our multivariable model, 7.45, compared women who did and did not report experiencing pressure from a health professional for cesarean birth, with experience of pressure strongly associated with having an unplanned primary cesarean. While provider pressure was likely, in some cases, the result of a concern with a medical condition, this was a low-risk population and variables for pregnancy complications and labor and birth complications (one or more item selected from either category of the participant’s birth certificate) were included as covariates in the model.

There is strong rationale, from *Listening to Mothers* survey data and other sources, for including the pressure variable in our model and thus concluding that many women experience pressure for cesarean birth that is not synonymous with provider recommendations for indicated cesarean birth. First, many women with unplanned primary cesareans did not report experiencing pressure, suggesting that they concurred that cesarean was an appropriate clinical decision. Second, rates of cesarean for subjective indications (e.g., non-reassuring fetal heart tracings, arrest of dilation, suspected large baby) vary widely, have contributed to the trend of increased national cesarean rates, and suggest a considerable amount of discretion in commonly identified indications [50]. Notably, a secondary analysis of this item in the third national *Listening to Mothers* survey found that women who had cesareans without medical indication were more likely to report experiencing pressure than women with cesareans for standard indications [51]. Another secondary analysis of data from that survey found that providers’ discussions of mode of birth among women with one or two prior cesareans pushed the decisions toward repeat cesareans in providing strikingly more information in support of having a cesarean rather planning a vaginal birth and in providers’ tendency to recommend repeat cesarean, suggesting poor conformity with standards of shared decision making [52]. Another secondary analysis from the same survey found that women’s reported perception of experience of pressure was highly associated with breakdowns in communication, specifically among the four in ten women who reported having held back from asking questions due to perceiving that clinicians were rushed, awareness of a discrepancy between their own care preferences and that of their providers, and/or fear of being perceived as difficult [53].

Given broad recognition that many cesareans can and should be safely avoided and widespread agreement about the importance of safely lowering the cesarean rate in many settings [2–4], it is likely that many women who reported feeling “pressure from any health professional” to have a cesarean experienced this as coercion and suspected the procedure may not have been needed. This was confirmed in some open-ended comments we received when asking about the worst aspect of participants’ hospital experience. Examples include “I felt rushed to deliver or else have a c-section,” the worst part was “encouraging c-section,” and “I didn’t like that they pressured me into having a c-section when I clearly wanted a natural birth. There was nothing wrong with my baby.” By contrast, we believe that many women who concurred with professional guidance to have a cesarean birth would have understood their provider’s position as a recommendation reflecting wise judgment and not “pressure.” For example, one participant wrote, “I personally felt pressured to have a C-section at first until they told me what the baby weighed. Then I was ok with it.”

The revision and validation of the Labor Culture Survey was carried out among 110 California hospitals, and found that the hospital unit NTSV rate over 2015–2016 was associated with a series of dimensions of hospital maternity unit culture, such as unit microculture, fear of vaginal birth, physician oversight, and maternal agency [27]. Bivariately, we compared participants’ birth hospital units meeting and not meeting the national consensus NTSV target rate of 23.9%, and found that our survey participants’ likelihood of having an unplanned primary cesarean trended toward their birth hospital unit not meeting the NTSV target, although this did not reach significance. In the multivariable model, hospitals in the highest quartile trended toward significantly higher cesarean rates, suggesting a potential role for hospital unit culture; however, we again did not find significant differences among the quartiles.

Areas for future research include examining the relationship between labor practices and unplanned primary cesarean birth with larger samples and studies that better capture timing and rationale for use of the practices. There is also a need to better understand ways to increase women’s agency and engagement in their care to the extent that their values and preferences affect the care they receive. Lastly, the small number of multiparous mothers who, by definition had given birth vaginally in the past and this time experienced a cesarean, were particularly likely to report having experienced pressure. This may be a productive area for future research on cesarean decision making with larger samples.

Limitations of our study include lack of power, as discussed above, to measure some labor management practices that have clearly been associated with cesarean birth in other studies. The relatively small number of multiparous mothers in our sample who had a primary cesarean also limited our ability to analyze their experiences. We also did not have information concerning timing of decisions that would allow a more nuanced analysis. For example, we could not determine whether midwives provided care during labor prior to an obstetrician-attended cesarean birth and which maternity care provider made the decision for a cesarean, nor could we distinguish epidural use for pain relief during labor versus its initiation as an anesthetic for surgery. We were unable to determine whether women who entered the hospital with less cervical dilation might have been experiencing less productive labor. Although previous analyses of survey questions about experiencing pressure to have interventions suggest that experience of “pressure” discriminates from concurring that provider recommendations are indicated, we have not systematically asked survey participants about their interpretation of these items. We also did not know the extent to which the index birth experience influenced women’s postpartum attitude toward intervention in childbirth and whether that attitude was held prior to the birth. Further, this secondary analysis of a cross-sectional design may not have adjusted for some relevant confounders, including some related to risk and complications. For example, we adjusted for any pregnancy and labor and birth complication identified on participants’ birth certificates, yet cannot be certain that this corrected for early hospital admission or labor induction associated with conditions that increase the likelihood of cesarean birth.

## Conclusions

In the present context of overuse of cesarean birth in the United States and most other nations, women’s beliefs, preferences, knowledge and behaviors did not appear to impact their mode of birth. Additional support, educational programs and engagement strategies may be needed to enable more reliable translation of their preferences into actual care received. Such improvements should include attention to better communication and decision-making processes. Meanwhile, changes in hospital labor management provide clear opportunities for reducing cesarean overuse and associated costs, improving outcomes and providing care more concordant with women’s wishes. The strong relationship between pressure and unplanned cesareans raises concerns about contemporary maternity care practice.

## Supplementary information

**Additional file 1.** LTM-CA Questionnaire_FINAL_8.27.18.pdf. *Listening to Mothers in California*: Survey Questionnaire. Complete questionnaire, in English and Spanish, used in the *Listening to Mothers in California* Survey

## Data Availability

A de-identified version of the *Listening to Mothers in California* Survey dataset analyzed during the current study has been uploaded into the Odum Institute Dataverse public data repository at the University of North Carolina (https://dataverse.unc.edu/dataverse/listeningtomothers, doi:10.15139/S3/3KW1DB), where it joins previous national *Listening to Mothers* survey datasets. For their analyses, study investigators have access to selected items from participants’ birth certificates and aid code, aid code category and program/plan code analysis variables from a state database relating to Medi-Cal (public assistance) beneficiaries. However, the California Department of Public Health prohibits investigators from including any items derived from birth certificates in a public dataset. Despite many requests and discussions, investigators did not receive a response to a request to include the identification of Medi-Cal beneficiary used in all investigator analyses (i.e., a paid 2016 childbirth claim in the state Medi-Cal database) in the public dataset. Public dataset users have access to participants’ survey response to the source of payment item to identify Medi-Cal beneficiaries (Kappa score on agreement of the two sources is 0.81). From the initial IRB protocol submission, investigators clearly disclosed their intent to make public a de-identified dataset from the survey. The IRB guidance to investigators about elements of informed consent required for this low-risk study did not include disclosure of this intent to make the dataset public, and IRB-approved outreach documents did not include that information in study informed consent processes, described above. The IRB approved an amendment requesting permission to transmit the dataset to the public repository. Many resources relating to the *Listening to Mothers in California* Survey are available at both http://www.nationalpartnership.org/LTMCA and http://www.chcf.org/listening-to-mothers-CA. National survey resources and a bibliography of *Listening to Mothers* reports from the core investigators and others are available at http://www.nationalpartnership.org/listeningtomothers.

## Abbreviations

aOR: Adjusted odds ratio
BMI: Body mass index
CA: California
CI: Confidence interval
cm: Centimeter
IRB: Institutional Review Board
n: Number
NTSV: Nulliparous term singleton vertex (cesarean)
OR: Odds ratio
p: Probability
ref: Reference
UCSF: University of California San Francisco
